# The first case of 38,XX (*SRY*-positive) disorder of sex development in a cat

**DOI:** 10.1186/s13039-015-0128-5

**Published:** 2015-03-26

**Authors:** Izabela Szczerbal, Monika Stachowiak, Stanislaw Dzimira, Krystyna Sliwa, Marek Switonski

**Affiliations:** Department of Genetics and Animal Breeding, Poznan University of Life Sciences, Poznan, Poland; Department of Pathology, Wroclaw University of Environmental and Life Sciences, Wroclaw, Poland; Veterinary Practice, Swarzedz, Poland

**Keywords:** Cat, Disorder of sex development, DSD, Intersexuality, SRY, Translocation X-Y, X chromosome inactivation

## Abstract

**Background:**

*SRY*-positive XX testicular disorder of sex development (DSD) caused by X;Y translocations was not yet reported in domestic animals. In humans it is rarely diagnosed and a majority of clinical features resemble those which are typical for Klinefelter syndrome (KS). Here we describe the first case of *SRY*-positive XX DSD in a tortoiseshell cat with a rudimentary penis and a lack of scrotum.

**Results:**

Molecular analysis showed the presence of two Y-linked genes (*SRY* and *ZFY*) and a normal sequence of the *SRY* gene. Application of classical cytogenetic techniques revealed two X chromosomes (38,XX), but further FISH studies with the use of the whole X chromosome painting probe and BAC probes specific to the Yp chromosome facilitated identification of Xp;Yp translocation. The *SRY* gene was localised at a distal position of Xp. The karyotype of the studied case was described as: 38,XX.ish der(X)t(X;Y)(p22;p12)(SRY+). Moreover, the X inactivation status assessed by a sequential R-banding and FISH with the *SRY*-specific probe showed a random inactivation of the derivative X^SRY^ chromosome.

**Conclusions:**

Our study showed that among DSD tortoiseshell cats, apart from XXY trisomy and XX/XY chimerism, also *SRY*-positive XX cases may occur. It is hypothesized that the extremely rare occurrence of this abnormality in domestic animals, when compared with humans, may be associated with a different organisation of the Yp arm in these species.

## Background

Sex chromosome abnormalities are well-known causes of disorders of sex development (DSD) in mammals [1]. In cats XXY trisomy is the most frequently reported abnormality [2]. Identification of this trisomy in cats is easy due to the tortoiseshell coat colour, which is unusual in male cats. Since the gene encoding the orange coat colour is located on the X chromosome, a tortoiseshell pattern, caused by a random X-inactivation, is typical of heterozygous female cats. Also in the human XXY trisomy (Klinefelter syndrome - KS) is the most common sex chromosome aneuploidy, with a prevalence of about 1 in 660 new born boys [3]. The most characteristic clinical features associated with KS include a tall stature, small azoospermic testes and gynecomastia [4]. Cats with XXY trisomy are also infertile, but clinical signs of this disorder vary [5].

The XX (*SRY*-positive) male syndrome, officially classified as 46,XX testicular DSD and also known as de la Chapelle syndrome, is rarely diagnosed in humans – 1 in 20 000 newborn boys [6]. This syndrome is caused by a translocation of a fragment of the Y chromosome, carrying the *SRY* gene, to the X chromosome during paternal meiosis [7]. Interestingly, the phenotype of this syndrome mimics KS to some extent, but major differences concern a lower body height and the presence of maldescended testes [6]. So far, to our best knowledge there have been no reports on *SRY*-positive XX DSD in domestic animals. In this report we describe the first case of a 38,XX tortoiseshell cat with Xp;Yp translocation and the presence of the *SRY* gene.

## Case presentation

**Figure 1:**
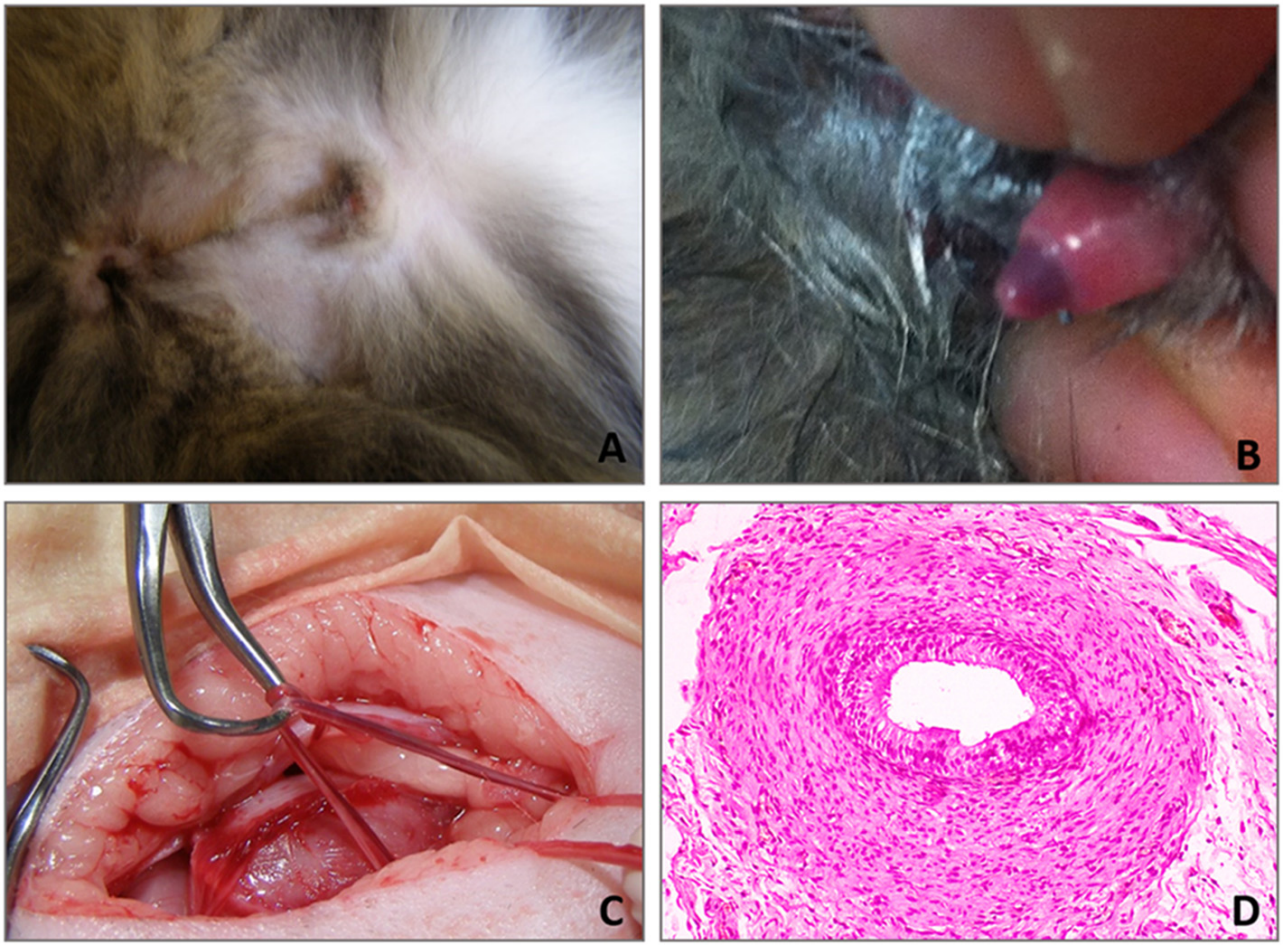
**Phenotype of the cat: A) external genitalia, B) underdeveloped penis, C) identification of ductus deferens during surgery, D) histology of the ductus deferens.**

An eight-month old tortoiseshell cat was presented for clinical examination to identify sex of the patient. The physical examination revealed a small body size, female appearance, no scrotum and the presence of an underdeveloped penis without visible spines (Figure 1A and B). The cat was presented for neutering. The surgery did not reveal the presence of the gonads, although ductuli deferentes were identified and histological examination confirmed their normal organization (Figure 1C and D).

## Results

**Figure 2:**
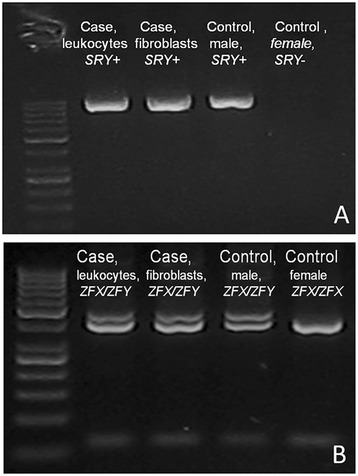
**Molecular detection of** ***SRY*** **and** ***ZFX/ZFY*** **genes. A)** The presence of the *SRY* gene amplicon in leukocytes and fibroblasts of the cat. *SRY* positive and *SRY* negative controls were included in the analysis, **B)** *ZFX* and *ZFY* genes were detected both in leukocytes and fibroblasts. *ZFX/ZFY* and *ZFX/ZFX* controls were included in the analysis.

The first step included a cytogenetic examination with the use of Giemsa staining and R-banding, as well as PCR searching for Y-linked genes (*SRY* and *ZFY*) in two cell lines, leukocytes and fibroblasts. A normal female chromosome complement (38,XX) and the presence of *SRY* and *ZFY* (along with the *ZFX* gene) were found in both cell lines (Figure 2). Sequencing of the *SRY* gene showed no variation (data not shown).

**Figure 3:**
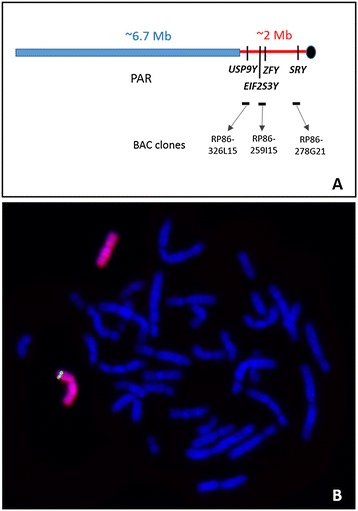
**Cytogenetic analysis. A)** Schematic presentation of the feline Yp arm, based on Li et al. [10], and location of the used BAC clones **B)** Dual-colour FISH with the use of whole X chromosome painting probe (red) and *SRY*-specific BAC probe (green).

Further cytogenetic analysis was carried out with the use of the FISH technique. A whole X chromosome painting probe, as well as a BAC probe harbouring the *SRY* gene (RP86-278G21) were used. The painting confirmed the presence of two X chromosomes, while the BAC probe hybridized to the short arm of a single X (Figure 3). In order to resolve whether other segments of Yp were also transmitted to the X chromosome, two additional Yp-specific BAC probes were used: RP86-326 L15 (harbouring *USP9Y*) and RP86-259I15 (harbouring *EIF2S3Y*). Both probes gave a specific hybridization signal in the distal part of a single X chromosome (Xp22). In addition, these probes hybridized to a pericentromeric region of Xp and the proximal part of Xq (data not shown). The same unspecific hybridization signal was observed in the X chromosome of a control male cat. The karyotype of the studied cat was described as 38,XX.ish der(X)t(X;Y)(p22;p12)(SRY+).

**Figure 4:**
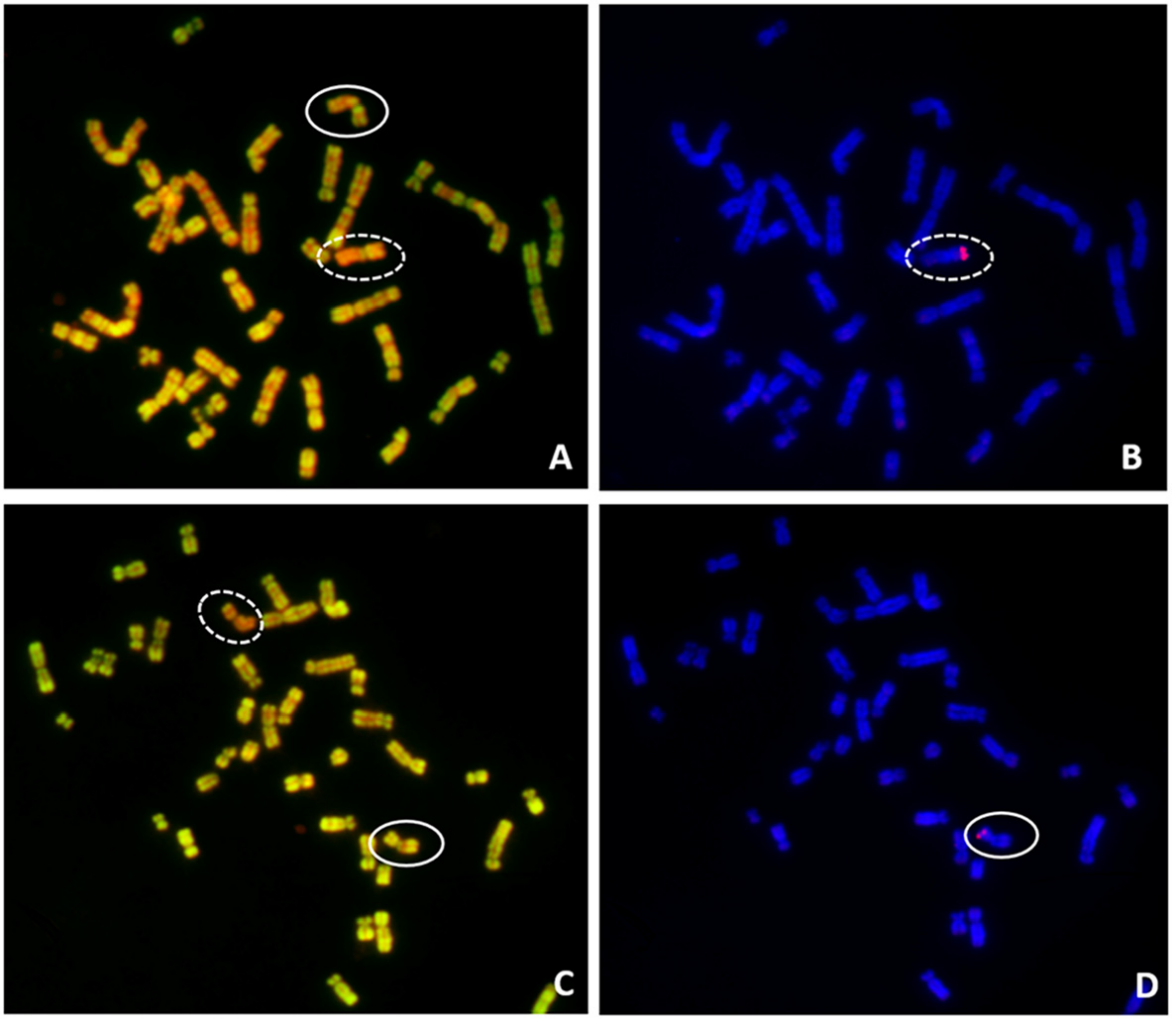
**Inactivation status of X and X** ^**SRY**^ **chromosomes. A) and C) R-banded metaphases, B) and D) FISH with** ***SRY*** **-specific BAC probe (red) on the same DAPI-banded metaphases.** Active X chromosome is marked by a solid circle and inactive by a doted circle.

Finally, we analyzed the activation/inactivation status of the X chromosome carrying the *SRY* gene with the use of the *SRY*-specific BAC probe on R-banded metaphases (Figure 4). We observed the hybridization signal on the active X, as well as inactive derivate X^SRY^ chromosomes (53% *versus* 47%). Thus, we assumed that the X^SRY^ derivate was randomly inactivated.

## Discussion

The tortoiseshell coat pattern in male cats suggests a disorder of sex development, related with the presence of two or more X chromosomes. An extensive questionnaire survey comprising data of 4598 male cats revealed that tortoiseshell cats occur rarely - 0.4% [8]. Eleven of these tortoiseshell males were cytogenetically studied and the most common sex chromosome complement was XX/XY chimerism (6 cases), followed by XXY trisomy (2 cases), XY (1 cases) and XX (2 cases) [8]. Identification of the XY complement may indicate that another cell line (XXY or XX) was not detected due to its low incidence. In the case of the XX complement one can hypothesize different scenarios: the presence of a minor cell line (XXY or XY), *SRY*-negative XX DSD or *SRY*-positive XX DSD.

Our study showed for the first time that transition of a fragment of the Y chromosome, harbouring the *SRY* gene, to the X chromosome is responsible for *SRY*-positive XX DSD in this species. Elucidation of the mechanism responsible for the reported mutation and identification of the break point on feline Yp is not easy, due to its unique organisation, in terms of gene order and size of the pseudoautosomal region (PAR) [9,10]. In the feline Yp the PAR is large (approx. 6.7 Mb) and the distance between the *SRY* gene and PAR is approx. 1.7 Mb [10]. As a consequence, the *SRY* gene is located in the proximal half of feline Yp. On the gene map of feline Yp only two multicopy genes (*CYorf15* and *HSFY)* are located more proximally and their copies are present on both sides of the *SRY* gene [10]. Thus, PCR detection of these genes is not useful in searching for the break point. It has been suggested that a non-allelic homologous recombination (NAHR) is responsible for the X;Y translocation in the human and it was indicated that the NAHR events preferentially happen around the *PRKY* pseudogene [11]. Unfortunately, there is no information available concerning the orthologous sequence in the cat Y chromosome.

The X inactivation pattern in *SRY*-positive XX DSD cases, in relation to their phenotype, is still under discussion. Some authors hypothesized that preferential inactivation of X^SRY^ is related with undermasculinization [12]. However, other reports did not confirm this relationship [13]. The studied cat showed a random inactivation pattern and pronounced undermasculinization, and thus our result is in agreement with the latter opinion.

In the recent years an increased number of sex chromosome abnormalities and DSD cases have been reported in cats. Apart from XXY trisomy [5], also X monosomy was described [14]. Among animals with a normal complement of sex chromosomes the *SRY*-positive XY DSD form was predominant. These animals had variable phenotypes: urogenital or anogenital congenital abnormalities [15,16], hypospadias [17], gynecomastia, presence of penis and an empty scrotum [18], ovotestes and Mullerian and Wolffian duct derivatives [19]. On the other hand, no XX DSD cases were reported [2]. Thus, the presented case (*SRY*-positive XX DSD) may be considered very rare. The phenotype of this cat (small body size, underdeveloped penis and gonadal dysgenesis) resembled major clinical characteristics of human *SRY*-positive XX DSD (short stature, cryptorchidism and micropenis).

## Conclusions

Two major forms of testicular/ovotesticular XX DSD are recognized in mammals: *SRY*-positive and *SRY*-negative. The most common form diagnosed in the human is *SRY*-positive [20], while in domestic animal species (dog, pig, goat, horse) the *SRY*-negative form has only been described [1]. We hypothesize that the probability of *SRY* translocation on Xp may depend on a different organisation (size of PAR, distance between *SRY* and PAR, gene order, etc.) of the Yp arm in the human and domestic mammals.

## Methods

### Histological analysis

Tissue samples were fixed with 10% formalin. Tissue sections of 3–5 μm in thickness were stained using the Masson–Goldner method (haematoxylin and eosin) and analyzed under an Olympus CX41 light microscope equipped with Olympus digital camera.

### Molecular analysis

DNA was isolated from a blood sample (leukocytes ) collected on EDTA and fibroblasts culture with the use of commercial kits (Blood Mini and Genomic Mini, respectively, A&A Biotechnology, Poland). The Y-linked genes were detected by PCR (SRY) and PCR-RFLP (ZFX/ZFY). PCR conditions, primer sequences and the used restriction enzyme were described earlier by [14].

The *SRY* amplicon was purified with Exonuclease I (Thermo Scientific, USA) and Thermosensitive Alkaline Phosphatase (Thermo Scientific, USA) sequenced with the use of a BigDye® Terminator v3.1 Sequencing Kit (Applied Biosystems; USA) and analyzed on an ABI 3130 Genetic Analyzer (Applied Biosystems; USA).

### Conventional cytogenetic analysis

Chromosome preparations were obtained from skin fibroblast cultures. Cytogenetic evaluation was carried out with the use of conventional Giemsa staining and R-banding according to the procedures described by Iannuzzi and Di Berardino [21]. Cat chromosomes were identified based on karyotype nomenclature proposed by Cho et al. [22].

### Fluorescence in situ hybridization (FISH)

The locus-specific BAC probes were obtained from the Feline BAC library RPCI-86 (https://bacpac.chori.org/). The following clones - RP86 278G21, RP86-326 L15 and RP86-259I15, specific to the short arm of the Y chromosome were selected, based on [23,24] The whole feline X chromosome painting probe was obtained by flow sorting (kindly provided by Prof. M.A. Ferguson-Smith, Cambridge University, UK). DNA was labelled with biotin-16-dUTP or digoxigenin-11-dUTP by random priming (BAC clone) or DOP-PCR (painting probes) and the standard protocol for FISH experiments was applied, as described earlier by Szczerbal et al. [25]. Microscopic evaluation was carried out under a Nikon E600 Eclipse fluorescent microscope (Melville, NY, USA), equipped with a cooled CCD digital camera and Lucia software.
